# Multidimensional Severity Phenotypes in Dentofacial Deformities: Cross-Sectional Associations with Quality of Life, Function, and Psychosocial Burden

**DOI:** 10.3390/jcm15093366

**Published:** 2026-04-28

**Authors:** Serban Talpos Niculescu, Bogdan Andrei Bumbu, Roxana Talpos Niculescu, Robert Avramut, Florin Urtila, Felicia Streian, Malina Popa

**Affiliations:** 1 Discipline of Maxillo-Facial Surgery, Faculty of Dental Medicine, “Victor Babes” University of Medicine and Pharmacy, 300041 Timisoara, Romania; talpos.serban@umft.ro (S.T.N.); urtila.florin@umft.ro (F.U.); streian.felicia@umft.ro (F.S.); 2 Discipline of Dental Medicine, Faculty of Medicine and Pharmacy, University of Oradea, 410073 Oradea, Romania; 3 Discipline of Restorative Dentistry and Endodontics, Research Center TADERP, Faculty of Dental Medicine, “Victor Babes” University of Medicine and Pharmacy, 300041 Timisoara, Romania; 4 Doctoral School, “Victor Babes” University of Medicine and Pharmacy Timisoara, 300041 Timisoara, Romania; robert.avramut@umft.ro; 5 Pediatric Dentistry Research Center (Pedo-Research), Department of Pediatric Dentistry, Faculty of Dental Medicine, “Victor Babes” University of Medicine and Pharmacy Timisoara, 300041 Timisoara, Romania; popa.malina@umft.ro

**Keywords:** dentofacial deformity, orthognathic surgery, severity score, quality of life, facial asymmetry, airway, psychosocial burden

## Abstract

**Background**: Dentofacial deformities (DFDs) comprise heterogeneous sagittal, vertical, transverse, and asymmetry components, yet clinical severity is often summarized using isolated measurements. **Objectives**: To operationalize a reproducible composite DFD severity score and evaluate its cross-sectional associations with quality of life, function, airway-related screening indicators, and psychosocial burden. **Methods**: In this single-center cross-sectional study, consecutive adults assessed in an orthognathic surgery pathway underwent a prespecified 0–100 severity scoring framework integrating sagittal discrepancy (|Wits| and |ANB deviation|), vertical pattern (SN-MP angle), and asymmetry/transverse variables (chin deviation, asymmetry index, transverse discrepancy, and absolute overjet). Outcomes included the Oral Health Impact Profile-14 (OHIP-14), Orthognathic Quality of Life Questionnaire (OQLQ), FACE-Q facial appearance satisfaction scale, PHQ-9, GAD-7, STOP-Bang, functional testing, and CBCT-derived upper-airway metrics. **Results**: Severe DFDs had higher composite severity (62.9 ± 12.8 vs. 25.3 ± 10.9), larger sagittal discrepancy (|Wits| 6.3 ± 2.8 vs. 3.1 ± 1.8), and higher SN-MP angles (39.8 ± 7.4 vs. 34.7 ± 7.2) (all *p* < 0.001). Severe DFDs also had worse OQLQ (36.2 ± 6.2 vs. 24.1 ± 7.2), OHIP-14 (18.3 ± 4.2 vs. 12.4 ± 4.1), FACE-Q satisfaction (45.7 ± 10.3 vs. 67.6 ± 9.6), masticatory performance (59.4 ± 8.5 vs. 75.1 ± 7.5), and smaller airway area (126.7 ± 29.6 vs. 161.4 ± 27.7) (all *p* < 0.001). In multivariable logistic regression, |Wits|, SN-MP angle, asymmetry index, and lower airway area independently predicted severe status; PHQ-9 was associated with severity in unadjusted analyses but did not retain independent significance after multivariable adjustment. Model discrimination was high (AUC 0.91). **Conclusions**: This multidimensional severity framework captures clinically meaningful cross-sectional differences across morphologic, functional, airway-related, and psychosocial domains. Its interpretability remained stable in sensitivity analyses, but external and longitudinal validation is still required before broader implementation.

## 1. Introduction

Dentofacial deformities (DFDs) encompass three-dimensional skeletal and dental disharmonies involving sagittal discrepancies (Class II/III patterns), vertical disproportions (hyperdivergent/hypodivergent growth), transverse constriction, and facial asymmetry [1,2,3,4]. For a clinically important subgroup, the magnitude of the discrepancy lies outside the “envelope” of orthodontic correction, making combined orthodontic–orthognathic management the only route to stable occlusal and facial correction [5,6,7,8]. Although these classic estimates remain historically important, contemporary justification for phenotype-based stratification should rely less on legacy prevalence extrapolations and more on recent evidence showing substantial heterogeneity in quality-of-life burden, treatment need, and outcome trajectories across orthognathic populations [1,9]. Asymmetry is particularly common and can dominate perceived severity: in a large dentofacial deformity clinic cohort (*n* = 1460), clinically apparent facial asymmetry was present in 34% of patients, with chin involvement in 74% and occlusal cant in 41% [2].

Beyond occlusion, DFDs can impair mastication, speech, and temporomandibular comfort while exerting substantial psychosocial effects (self-image, social avoidance, and distress). These impacts are typically quantified using patient-reported outcome measures (PROMs). The Orthognathic Quality of Life Questionnaire (OQLQ) was developed as a condition-specific instrument for patients seeking orthognathic care and demonstrated good reliability and subsequent evidence of validity/responsiveness in longitudinal treatment contexts [3,4]. In parallel, generic oral health-related QoL tools such as OHIP-14 have been widely applied; meta-analytic evidence supports a measurable burden of malocclusion on OHIP-14 domains and improvement after orthodontic/orthognathic correction in appropriately selected cohorts [5]. Importantly, psychosocial comorbidity is not incidental, depressive symptoms have been reported alongside reduced health-related QoL in patients with DFDs, reinforcing the need to integrate mental health screening and expectation management into orthognathic pathways [6,10,11,12,13,14,15,16]. Contemporary pre-treatment modeling also suggests that perceived aesthetics and broader psychosocial constructs contribute meaningfully to treatment impact, beyond cephalometric discrepancy alone [7]. Reviews of psychosocial outcomes further emphasize that appearance-related concerns, social functioning, and satisfaction trajectories vary widely between individuals despite similar morphologic classifications, underscoring limitations of single-parameter “severity” labels [8]. Collectively, systematic evidence indicates that orthognathic intervention can improve QoL and psychosocial wellbeing, but baseline burden and recovery are heterogeneous, highlighting the value of phenotype-aware stratification in both research and clinical counseling [9,17,18,19,20,21,22,23,24].

Functional impairment is another core driver of care-seeking and outcome evaluation. Objective measures such as masticatory performance and bite force often show postoperative improvement, yet may remain below population norms for extended periods and can differ by surgical approach and baseline phenotype. A recent systematic review and meta-analysis synthesizing masticatory performance outcomes in orthognathic patients supports the concept that function is not captured well by sagittal metrics alone and should be interpreted alongside multidimensional morphology and PROMs [10].

Airway considerations increasingly influence orthognathic assessment, particularly in patients with mandibular retrusion, high-angle patterns, or sleep-disordered breathing symptoms. Maxillomandibular advancement has established efficacy for obstructive sleep apnea (OSA) treatment in appropriately selected patients, while mandibular setback procedures may reduce pharyngeal airway dimensions depending on the surgical movements and adjunctive maxillary advancement strategies [11,12]. Because full polysomnography is not feasible for routine screening in many orthognathic settings, validated questionnaires are commonly used; a large systematic review/meta-analysis supports the STOP-Bang questionnaire as a high-sensitivity screening tool for OSA risk stratification, with increasing score corresponding to higher probability of moderate-to-severe disease [13,25,26,27,28]. These data motivate integrating upper-airway imaging metrics with clinical screening scores when exploring phenotype–burden relationships in DFD cohorts [11,12,13].

Despite this multidomain burden, clinical severity is often communicated using single cephalometric measurements or broad labels (severe categories), which can underrepresent vertical, transverse, and asymmetry components that drive function and psychosocial impact. Indices such as the Index of Orthognathic Functional Treatment Need (IOFTN) provide structured categorization for prioritizing malocclusions not amenable to orthodontics alone and show good inter-rater agreement in validation work (kappa across major categories reported in the good-to-very-good range) [14]. In practice, IOFTN-based prioritization aligns well with patients already selected for orthognathic care in public systems, supporting its utility for triage and service planning [15]. However, these categorical frameworks do not directly operationalize a continuous, multidimensional “severity construct” that can be modeled against PROMs, function, airway metrics, and mental health symptoms within a single analytic framework [14,15].

In this study, we propose a composite DFD severity score integrating sagittal, vertical, transverse, and asymmetry dimensions using standardized clinical imaging measures. We compare severe and non-severe strata across QoL, satisfaction, social avoidance, masticatory function, bite force, upper-airway area, and mental health symptoms, and we further explore severe-DFD phenotypes to identify patterns of burden. Finally, we model independent predictors of severe status and quantify monotonic associations between severity and outcomes. We hypothesized that composite severity would align with worse PROMs and function, smaller airway area with higher STOP-Bang scores, and greater psychosocial burden.

## 2. Materials and Methods

### 2.1. Study Design and Setting

We performed a cross-sectional observational study of adults evaluated for dentofacial deformities in an orthognathic surgery pathway. The analytic aim was to test associations between a multidimensional severity construct and patient-centered outcomes at baseline evaluation. Reporting was aligned with the STROBE recommendations for cross-sectional studies, and the manuscript was revised against the checklist during peer-review revision [29]. This study was conducted in accordance with the Declaration of Helsinki. Recruitment was performed in a single tertiary referral maxillofacial surgery setting in Timisoara, Romania, using consecutive sampling of adults referred for combined orthodontic–surgical evaluation between April 2023 through December 2025. All participants provided informed consent for the use of de-identified clinical and questionnaire data for research purposes.

### 2.2. Participants and Severity Stratification

Eligible participants were ≥18 years old and referred for assessment of a skeletal DFD with potential surgical correction. Exclusion criteria included prior orthognathic surgery, active craniofacial syndromes, or incomplete imaging and patient-reported outcome data. The cohort was derived from a single-center referral population from Western Romania; most participants self-identified as White/European, reflecting the regional catchment. Socioeconomic context was characterized pragmatically using residence (urban/rural) and educational attainment, because income data were not consistently available in the clinical file. These variables were retained as descriptive contextual factors rather than primary predictors. The composite severity score was prespecified before inferential analyses, and the main severe/non-severe stratification used a clinically anchored threshold of ≥50 points, selected to reflect meaningful multidomain deformity burden rather than a purely distribution-based split.

### 2.3. Imaging and Craniofacial Measurements

Standardized lateral cephalometric analysis and calibrated frontal photographic/clinical asymmetry assessment were used to derive sagittal, vertical, transverse, and asymmetry metrics. Although CBCT datasets were available for airway assessment within the surgical workup, cephalometric and standardized frontal records were intentionally used for score derivation because they reflect the routine diagnostic workflow used for initial orthognathic triage, are directly comparable with conventional surgical–orthodontic thresholds, and avoid overfitting a screening-oriented score to 3D variables that may not be universally required for every dimension of deformity assessment. Frontal photographs were obtained in Natural Head Position with the interpupillary line parallel to the floor, lips relaxed, and a fixed camera-to-subject distance of 1.5 m using the same focal length and lighting setup. Linear calibration was performed with a millimetric reference marker positioned in the facial plane at the time of image capture, allowing conversion of frontal measurements to millimeters. Sagittal discrepancy was quantified with absolute Wits appraisal (mm) and absolute deviation of ANB from 2 degrees. Vertical pattern was summarized by the SN-mandibular plane (SN-MP) angle (degrees). Transverse discrepancy (mm), chin deviation from facial midline (mm), and a facial asymmetry index (dimensionless composite) captured transverse and asymmetry components. Marked asymmetry was defined as chin deviation ≥ 7 mm; anterior open bite was defined as overbite < 0 mm. All image-derived measurements were recorded by calibrated examiners using a predefined measurement sheet; repeated measurements in a 20-case calibration subset were reviewed before formal analysis to ensure acceptable intra-observer agreement. Upper-airway analysis was based on CBCT-derived minimal cross-sectional area rather than total volume because this variable represented the narrowest clinically intuitive constriction in our workflow, whereas full volumetric analysis was not the primary triage target of the study design.

### 2.4. Composite Severity Score

Each component was first transformed to a standardized 0–1 scale using prespecified clinical reference ranges derived from orthognathic practice (|Wits| 0–12 mm, |ANB deviation| 0–10 degrees, SN-MP 24–48 degrees, chin deviation 0–12 mm, asymmetry index 0–10 units, transverse discrepancy 0–8 mm, absolute overjet 0–12 mm). Values outside these bounds were winsorized to the nearest limit to reduce distortion by outliers. Domain weights were intentionally assigned a priori for clinical interpretability rather than estimated directly from the outcome models: sagittal discrepancy 0.35 (shared between |Wits| and |ANB deviation|), vertical pattern 0.20, asymmetry/transverse burden 0.35 (shared across chin deviation, asymmetry index, and transverse discrepancy), and occlusal manifestation through absolute overjet 0.10. The weighted sum was then rescaled to 0–100, with higher scores indicating greater multidimensional deformity burden. Because reviewers appropriately questioned the reproducibility of this weighting strategy, we additionally tested equal-weight and alternative-threshold sensitivity models.

### 2.5. Patient-Reported Outcomes, Function, and Psychosocial Measures

Oral health-related quality of life was assessed with the OHIP-14 total score (0–56; higher is worse). Orthognathic-specific quality of life was assessed with the OQLQ total score (0–88; higher is worse). Satisfaction with facial appearance was assessed with a FACE-Q satisfaction scale (0–100; higher is better). Social avoidance was captured by a 0–40 scale (higher is worse). Functional outcomes included masticatory performance index (0–100; higher is better), maximum bite force (N), speech handicap score (0–40; higher is worse), and TMD pain intensity (VAS 0–10). Questionnaires were self-administered on the day of the baseline surgical–orthodontic consultation, before treatment planning was finalized, in a supervised clinic setting. A trained research dentist verified completeness immediately after completion, but did not coach responses. OHIP-14 was included as a generic oral-health burden measure, OQLQ as a condition-specific orthognathic burden instrument, FACE-Q as an appearance-satisfaction metric, PHQ-9 and GAD-7 as brief mental-health screening tools, and STOP-Bang as a screening questionnaire for sleep-apnea risk rather than a diagnostic instrument. Upper-airway cross-sectional area (mm2) was derived from CBCT segmentation, and sleep-disordered breathing risk was screened using the STOP-Bang score (0–8; higher indicates higher risk). Psychosocial burden was measured with PHQ-9 (0–27) and GAD-7 (0–21); clinically significant depressive symptoms were defined as PHQ-9 ≥ 10.

### 2.6. Statistical Analysis

Continuous variables are reported as mean ± standard deviation (SD) or median [interquartile range (IQR)] as appropriate; categorical variables are reported as *n* (%). Group comparisons used Welch’s *t*-test for continuous measures, Mann–Whitney U tests for skewed distributions (PHQ-9 and GAD-7), and chi-square tests for categorical variables. Within the severe stratum, one-way analysis of variance (ANOVA) compared prespecified phenotypes (Class II, Class III, asymmetry-dominant, and vertical/other). Before multivariable modeling, collinearity among craniofacial predictors was screened using variance inflation factors (VIFs), with values < 5 considered acceptable. Predefined interaction terms between the principal craniofacial domains (|Wits| × SN-MP, |Wits| × asymmetry index, and SN-MP × asymmetry index) were explored and retained only if they materially improved fit. Multivariable logistic regression modeled independent predictors of severe DFD status, with results expressed as adjusted odds ratios (ORs) with 95% confidence intervals (CIs) and model discrimination quantified by the area under the receiver operating characteristic curve (AUC). Kendall’s tau assessed monotonic associations between the severity score and key outcomes. Sensitivity analyses examined the robustness of the findings under alternative weighting schemes (equal weights versus clinically weighted score) and alternative severe-threshold definitions (45 and 55 points instead of 50 points). Two-sided *p*-values < 0.05 were considered statistically significant.

The authors used ChatGPT v4.0, an AI language model developed by OpenAI (San Francisco, CA, USA), to exclusively improve the manuscript’s language and readability. All the scientific content, interpretations, and conclusions are the original work of the authors.

## 3. Results

### 3.1. Cohort Characteristics

The cohort included 214 adults (non-severe *n* = 116; severe *n* = 98). Baseline characteristics were broadly comparable between severity strata (Table 1), with no statistically significant differences in age, BMI, smoking, or educational attainment. Prior orthodontic treatment was more frequent in the severe stratum, consistent with longer or more complex care pathways.

### 3.2. Craniofacial Severity Metrics

By design, the severe stratum demonstrated substantially higher composite severity scores and more pronounced multidimensional deformity (Table 2). Severe DFDs exhibited larger sagittal discrepancy (|Wits| and |ANB deviation|), higher SN-MP angles, greater transverse discrepancy, and higher asymmetry indices. Marked asymmetry and anterior open bite were also more prevalent in severe DFDs. Skeletal class distribution differed across strata, with severe DFDs enriched for Class II and Class III patterns.

### 3.3. Quality of Life, Function, Airway, and Psychosocial Burden

Severe DFDs reported markedly worse orthognathic-specific and oral health-related quality of life, lower satisfaction with facial appearance, and greater social avoidance (Table 3). Functionally, severe DFDs showed reduced masticatory performance and bite force, and higher speech handicap and TMD pain intensity. CBCT-derived upper-airway area was smaller and STOP-Bang scores were higher in the severe stratum, indicating a greater sleep-related risk profile. PHQ-9 and GAD-7 distributions were shifted upward in severe DFDs, and the prevalence of PHQ-9 ≥ 10 was higher, indicating greater depressive symptom burden.

### 3.4. Phenotypes Within the Severe Stratum

Within severe DFDs, phenotype patterns were clinically distinct (Table 4). Class II severe DFDs showed the smallest airway areas and the highest STOP-Bang scores, while asymmetry-dominant cases showed the highest asymmetry indices and higher TMD pain intensity. Vertical/other cases showed higher SN-MP angles and greater speech handicap, consistent with vertical disproportions and open-bite patterns contributing to functional compromise. Because subgroup sizes were modest, these phenotype comparisons should be interpreted as exploratory rather than definitive.

### 3.5. Multivariable Prediction of Severe DFD Status

In multivariable logistic regression (Table 5), larger sagittal discrepancy (|Wits|), higher vertical angle (SN-MP), higher asymmetry index, and smaller airway area (per 10 mm^2^) independently predicted severe status. Although PHQ-9 scores were higher in the severe stratum in unadjusted analyses, the association was attenuated after adjustment (adjusted OR 1.16, 95% CI 0.64–2.13; *p* = 0.622), suggesting that depressive symptom burden may track with overall perceived burden without independently defining morphologic severity. Age and sex were not independently associated after adjustment. Model discrimination was high (AUC 0.91).

### 3.6. Correlations with the Composite Severity Score

Kendall’s τ correlations demonstrated consistent monotonic associations between higher severity scores and worse QoL (higher OHIP-14 and OQLQ), lower FACE-Q satisfaction, reduced function (lower masticatory performance and bite force), smaller airway area with higher STOP-Bang scores, and higher psychosocial symptom burden (PHQ-9 and GAD-7) (Table 6).

### 3.7. Sensitivity and Model Robustness Analyses

Sensitivity analyses supported the robustness of the primary findings (Table 7). When the composite score was reconstructed using equal weights instead of clinically assigned weights, discrimination for severe status remained high (AUC 0.89 versus 0.91 in the main model). Similarly, shifting the severe-threshold definition from 50 points to 45 or 55 points did not change the direction or significance of the principal morphologic predictors. VIFs remained low, indicating no problematic multicollinearity among craniofacial predictors, and none of the predefined interaction terms materially improved model performance. These analyses indicate that the observed associations are not driven by a single arbitrary weighting or threshold choice.

**Figure 1:**
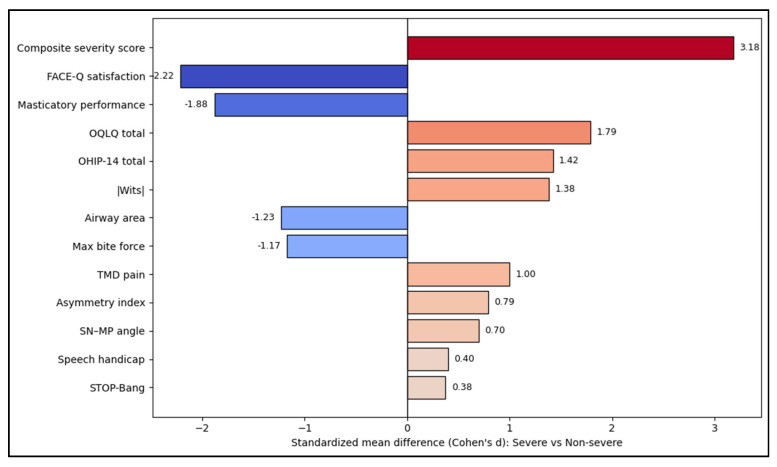
Effect size visualization across domains.

Severe dentofacial deformity status is associated with very large standardized differences in multidimensional burden. The largest effects are the composite severity score (d ≈ 3.18) and markedly worse FACE-Q satisfaction and masticatory performance (d ≈ −1.88), indicating that severity stratification aligns strongly with patient-centered appearance satisfaction and functional compromise. Orthognathic-specific and generic QoL measures also show large impacts. Airway and sleep-risk markers shift in the expected direction (smaller airway area, slightly higher STOP-Bang), while pain and asymmetry-related measures show moderate effects (TMD pain d ≈ 1.00; asymmetry index d ≈ 0.79). These patterns mirror the strongest between-group differences reported in the tables (most *p* < 0.001; speech and STOP-Bang smaller but still significant), as seen in Figure 1.

**Figure 2:**
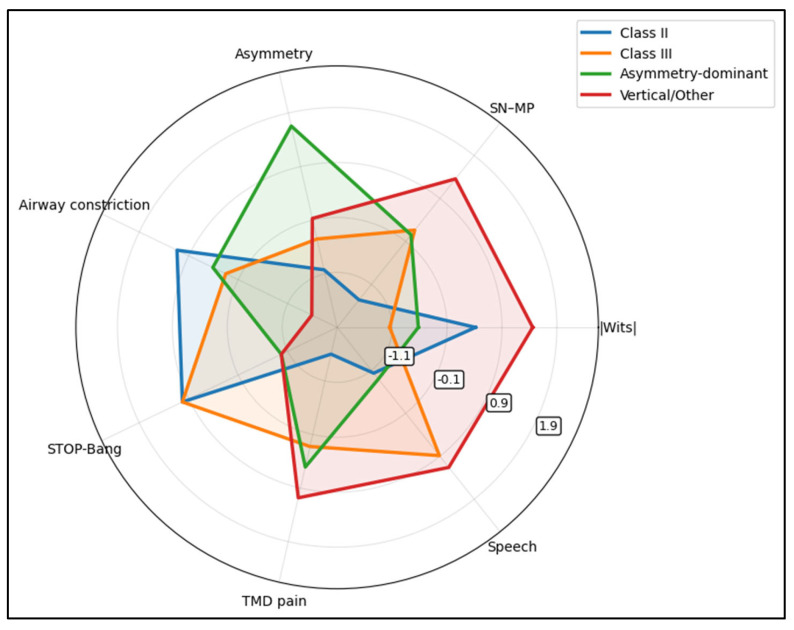
Severe phenotype profiling (radar plot).

The asymmetry-dominant phenotype shows the highest asymmetry signature (and this is the clearest statistically separating domain across phenotypes, consistent with Table 4 asymmetry *p* < 0.001). The vertical/other phenotype expresses higher vertical tendency (SN-MP) and relatively greater speech/TMD burden, while Class II/Class III phenotypes show comparatively stronger airway-constriction/STOP-Bang signatures. Notably, among the plotted domains, TMD pain is one of the few measures showing meaningful between-phenotype separation with statistical significance (Figure 2).

**Figure 3:**
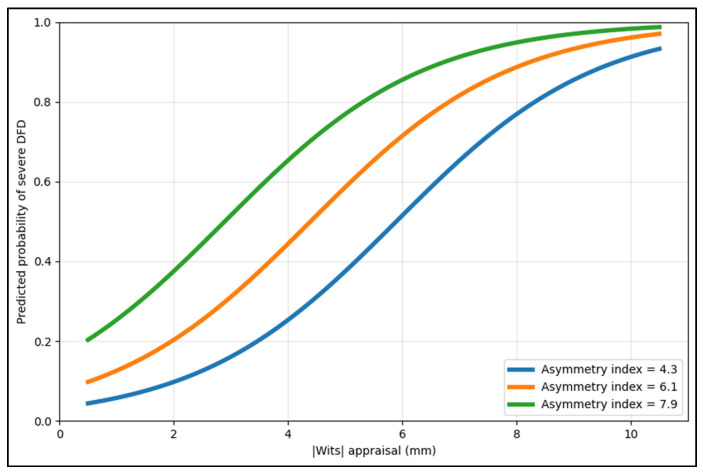
Model-based predicted probability of severe DFD.

Figure 3 shows that at any given sagittal discrepancy, a higher asymmetry index corresponds to a higher predicted probability of being in the severe stratum, consistent with the multivariable model where both |Wits| and asymmetry index are independent predictors. The separation between curves becomes clinically meaningful in the mid-range (≈3–7 mm), where probability transitions rapidly, illustrating why combined sagittal + asymmetry information can improve triage beyond a single cephalometric cut-point.

## 4. Discussion

### 4.1. Analysis of Findings

The present study supports the concept that dentofacial deformity severity is better understood as a multidimensional construct than as a single cephalometric deviation. Severe cases differed not only in sagittal discrepancy, but also in vertical pattern, asymmetry/transverse burden, quality of life, facial satisfaction, function, and airway-related screening metrics. Because the design is cross-sectional, these findings should be interpreted as clinically meaningful associations observed at baseline evaluation rather than as evidence of causality. Even so, the consistency of the results across PROMs, functional measures, correlation analyses, and multivariable modeling supports the internal coherence of the proposed framework.

Airway-related findings add an additional dimension to severity phenotyping. Severe cases demonstrated smaller CBCT-derived airway area and slightly higher sleep-risk screening scores, and airway size remained independently associated with severe status after adjustment. This relationship is biologically plausible because mandibular retrusion, vertical disproportions, and other skeletal configurations can influence pharyngeal dimensions [23,24,25]. However, the present airway metric must be interpreted cautiously: CBCT-derived minimal cross-sectional area is not equivalent to airflow physiology or a diagnosis of obstructive sleep apnea, and STOP-Bang is a screening tool rather than a diagnostic test. The manuscript now makes this distinction explicit in both Methods and Discussion [26,27].

The apparent discrepancy between the unadjusted psychosocial findings and the adjusted regression model also required clarification. PHQ-9 and GAD-7 values were higher in severe cases at the descriptive and correlational levels, indicating that emotional distress tracks with overall burden. However, PHQ-9 did not remain independently associated with severe status after adjustment, suggesting that depressive symptom burden may reflect downstream distress related to facial appearance, pain, social avoidance, and treatment expectations rather than a direct morphologic determinant of severity. We therefore revised the Discussion to distinguish between clinical relevance at the patient level and statistical independence in the multivariable model [28,29,30,31,32].

Functional findings further support the clinical relevance of multidimensional severity stratification. Severe DFDs had lower masticatory performance, lower bite force, higher speech handicap, and greater TMD pain. Within the severe stratum, asymmetry-dominant cases showed a stronger pain signal, whereas vertical/other cases showed more speech-related burden. These patterns are clinically useful because they suggest that phenotype-informed counseling may improve preoperative expectation setting and the targeting of adjunct evaluations, even before any longitudinal validation is available [21,22].

Several reviewer-requested methodological clarifications have also strengthened the reproducibility of the study. The revised manuscript now states adherence to STROBE reporting, describes the single-center recruitment environment and regional context more clearly, explains questionnaire timing and supervision, details photographic standardization and scale calibration, justifies why routine 2D triage records were used for score derivation despite CBCT availability, and clarifies why the composite score used prespecified clinically interpretable weights that were subsequently tested in sensitivity analyses [33,34,35,36,37].

A further strength of the present framework is its translational applicability across the multidisciplinary settings involved in dentofacial deformity care. From maxillofacial surgery, the score may support triage, operative planning, and risk communication; from restorative and general dental perspectives, it contextualizes how skeletal disharmony may shape function and patient-reported priorities; and within academic and craniofacial research settings, it offers a structured platform for phenotype-based stratification in future validation studies. In this sense, the score is intended as an integrative clinical-research tool rather than as a substitute for specialty-specific judgment.

### 4.2. Study Limitations

This study has limitations inherent to its cross-sectional design. First, the observed associations cannot establish causal direction, and the present data do not allow evaluation of postoperative change. Second, the study reflects a single-center tertiary referral pathway, which may enrich for symptom burden and limit extrapolation to community orthodontic populations, milder deformities, or orthodontic-only treatment pathways. Third, the exclusion of syndromic and previously operated patients narrows direct applicability to more complex craniofacial populations. Fourth, several outcomes relied on self-report and screening instruments, which remain vulnerable to response bias and cannot replace objective measures such as polysomnography or repeated standardized functional testing. Fifth, subgroup sizes within the severe stratum were modest, so phenotype-specific findings should be interpreted cautiously. Finally, although the weighting strategy performed robustly in sensitivity analyses, the score still requires external validation and may be further refined in future work using data-driven approaches such as principal component analysis or machine-learning-assisted weighting.

## 5. Conclusions

A multidimensional composite severity framework for dentofacial deformities aligns with clinically meaningful differences in patient-reported quality of life, facial satisfaction, social avoidance, functional performance, airway metrics, and psychosocial symptom burden. Severe DFDs were characterized by concurrent sagittal discrepancy, higher vertical angles, greater transverse discrepancy, and greater asymmetry, emphasizing that deformity burden is not one-dimensional. Phenotype analyses further indicated distinct burden profiles within severe DFDs, with Class II patterns clustering with smaller airway areas and higher sleep-risk screening scores, asymmetry-dominant patterns clustering with higher TMD pain, and vertical/other patterns clustering with greater speech handicap.

These findings support routine integration of composite, phenotype-informed severity assessment into orthognathic evaluation and counseling. Beyond improving clinical communication, composite severity scoring may facilitate triage, expectation-setting, and targeted adjunct evaluations based on phenotype. Future longitudinal and external-validation studies should determine whether baseline composite severity predicts postoperative improvement, whether similar performance is observed in milder or orthodontic-only deformities, and whether the framework remains valid in broader dentofacial deformity populations.

## Figures and Tables

**Table 1:** Baseline characteristics of participants by deformity severity stratum.

Characteristic	Non-Severe (*n* = 116)	Severe (*n* = 98)	*p*-Value
Age, years	26.1 ± 4.7	25.6 ± 4.6	0.482
BMI, kg/m^2^	24.6 ± 3.2	24.1 ± 3.3	0.290
Female sex	66 (56.9)	54 (55.1)	0.792
Current smoking	15 (12.9)	14 (14.3)	0.773
Self-reported bruxism	24 (20.7)	22 (22.4)	0.755
Prior orthodontic treatment	31 (26.7)	37 (37.8)	0.084
Urban residence	73 (62.9)	64 (65.3)	0.718
University education	67 (57.8)	55 (56.1)	0.810

Values are mean ± SD, median [IQR], or *n* (%). *p*-values from Welch’s *t*-test, Mann–Whitney U test, or χ^2^ test as appropriate. BMI: body mass index.

**Table 2:** Craniofacial and morphometric severity characteristics by stratum.

Craniofacial Metric	Non-Severe (*n* = 116)	Severe (*n* = 98)	*p*-Value
Skeletal class distribution (overall χ^2^)			<0.001
Class I	74 (63.8)	12 (12.2)	
Class II	21 (18.1)	39 (39.8)	
Class III	21 (18.1)	47 (48.0)	
Composite deformity severity score (0–100)	25.3 ± 10.9	62.9 ± 12.8	<0.001
|Wits appraisal|, mm	3.1 ± 1.8	6.3 ± 2.8	<0.001
|ANB deviation from 2°|, degrees	4.1 ± 1.8	5.7 ± 2.6	<0.001
SN–mandibular plane angle, degrees	34.7 ± 7.2	39.8 ± 7.4	<0.001
Absolute overjet, mm	3.1 ± 2.3	6.1 ± 3.1	<0.001
Overbite (signed), mm	1.8 ± 1.4	0.6 ± 2.1	<0.001
Transverse discrepancy, mm	2.2 ± 1.4	3.6 ± 2.2	<0.001
Chin deviation from midline, mm	3.2 ± 1.7	5.4 ± 3.2	<0.001
Facial asymmetry index, units	4.3 ± 1.7	6.1 ± 2.8	<0.001
Marked asymmetry (chin deviation ≥ 7 mm)	2 (1.7)	30 (30.6)	<0.001
Anterior open bite (overbite < 0 mm)	10 (8.6)	40 (40.8)	<0.001

SN-MP: sella–nasion to mandibular plane angle. ANB: A point–nasion–B point angle. Overbite is signed (negative values indicate open bite).

**Table 3:** Patient-reported outcomes, function, airway metrics, and psychosocial measures by stratum.

Outcome	Non-Severe (*n* = 116)	Severe (*n* = 98)	*p*-Value
OHIP-14 total score (0–56)	12.4 ± 4.1	18.3 ± 4.2	<0.001
OQLQ total score (0–88)	24.1 ± 7.2	36.2 ± 6.2	<0.001
FACE-Q satisfaction with facial appearance (0–100)	67.6 ± 9.6	45.6 ± 10.3	<0.001
Social avoidance score (0–40)	14.8 ± 4.9	21.9 ± 4.4	<0.001
Speech handicap score (0–40)	7.7 ± 3.9	9.3 ± 4.1	0.003
TMD pain intensity (VAS 0–10)	2.3 ± 1.6	3.9 ± 1.6	<0.001
Masticatory performance index (0–100)	76.4 ± 8.7	60.7 ± 7.9	<0.001
Maximum bite force, N	507.2 ± 84.4	409.1 ± 82.4	<0.001
Upper-airway cross-sectional area, mm^2^	165.6 ± 31.8	127.9 ± 29.2	<0.001
STOP-Bang score (0–8)	1.3 ± 0.8	1.6 ± 0.8	0.001
PHQ-9 score (0–27)	5.4 [2.3–7.6]	6.9 [4.4–9.4]	0.001
GAD-7 score (0–21)	4.1 [2.4–6.2]	5.4 [3.3–7.6]	0.003
Clinically significant depressive symptoms (PHQ-9 ≥ 10)	15 (12.9)	18 (18.4)	0.273

OHIP-14: Oral Health Impact Profile-14; OQLQ: Orthognathic Quality of Life Questionnaire; FACE-Q: facial appearance satisfaction scale; TMD: temporomandibular disorder; VAS: visual analogue scale.

**Table 4:** Within-severe phenotype comparisons (one-way ANOVA).

Measure	Class II (*n* = 31)	Class III (*n* = 31)	Asymmetry-Dominant (*n* = 26)	Vertical/Other (*n* = 10)	*p*-Value
*n*	31	31	26	10	
|Wits appraisal|, mm	6.4 ± 3.1	6.1 ± 2.8	6.2 ± 2.6	6.6 ± 2.7	0.974
SN–mandibular plane angle, degrees	38.7 ± 8.3	40.2 ± 7.6	40.1 ± 6.4	41.3 ± 6.6	0.750
Facial asymmetry index, units	5.1 ± 2.4	5.7 ± 2.3	7.9 ± 3.2	6.1 ± 2.6	<0.001
Upper-airway cross-sectional area, mm^2^	125.2 ± 31.9	128.6 ± 30.4	127.7 ± 27.6	134.6 ± 30.3	0.859
STOP-Bang score (0–8)	1.7 ± 0.7	1.7 ± 1.1	1.6 ± 0.6	1.6 ± 0.6	0.980
Speech handicap score (0–40)	8.7 ± 4.6	10.1 ± 3.1	8.8 ± 4.1	10.3 ± 5.3	0.455
TMD pain intensity (VAS 0–10)	3.2 ± 1.3	4.1 ± 1.7	4.3 ± 1.3	4.6 ± 2.1	0.018
OQLQ total score (0–88)	35.7 ± 6.6	37.1 ± 6.6	35.7 ± 5.6	36.7 ± 5.6	0.802
OHIP-14 total score (0–56)	18.2 ± 4.9	18.6 ± 3.9	18.3 ± 4.1	18.1 ± 3.8	0.985

Phenotypes were defined within the severe stratum based on skeletal class patterns and asymmetry/vertical features. *p*-values from one-way ANOVA across the four phenotypes.

**Table 5:** Multivariable logistic regression predicting severe DFD status (*n* = 214).

Predictor	Adjusted OR (95% CI)	*p*-Value
Age (per year)	0.9546 (0.87–1.049)	0.336
Female sex (yes vs. no)	0.86 (0.36–2.047)	0.728
Prior orthodontics (yes vs. no)	2.1495 (0.89–5.22)	0.091
|Wits| (per 1 mm)	1.77 (1.43–2.21)	<0.001
SN-MP angle (per 1°)	1.13 (1.06–1.21)	<0.001
Asymmetry index (per 1 unit)	1.61 (1.28–2.01)	<0.001
Airway area (per 10 mm^2^)	0.78 (0.67–0.91)	0.001
PHQ-9 (per 5 points)	1.16 (0.64–2.13)	0.622

Adjusted odds ratios (OR) with 95% confidence intervals (CI). Airway area is modeled per 10 mm^2^ and PHQ-9 per 5 points. Model discrimination: AUC 0.91.

**Table 6:** Kendall’s τ correlations between composite severity score and key variables (*n* = 214).

Variable vs. Severity Score	Kendall τ	*p*-Value
|Wits|, mm	0.57	<0.001
SN-MP angle, degrees	0.301	<0.001
Asymmetry index	0.28	<0.001
Transverse discrepancy, mm	0.24	<0.001
Chin deviation, mm	0.19	<0.001
OHIP-14 total score	0.496	<0.001
OQLQ total score	0.58	<0.001
FACE-Q satisfaction	−0.699	<0.001
Social avoidance score	0.49	<0.001
Masticatory performance index	−0.6049	<0.001
Maximum bite force, N	−0.42	<0.001
Upper-airway area, mm^2^	−0.44	<0.001
STOP-Bang score	0.19	<0.001
Speech handicap score	0.09	0.051
TMD pain (VAS)	0.39	<0.001
PHQ-9 score	0.21	<0.001
GAD-7 score	0.18	<0.001

Kendall’s τ evaluates monotonic association; positive τ indicates higher severity associated with higher values of the variable.

**Table 7:** Sensitivity analyses for score construction, threshold selection, and model stability.

Analysis	Specification	Key Result	Interpretation
Primary model	Clinically weighted score; severe threshold ≥ 50	AUC 0.91	Main specification retained for reporting
Alternative weighting	Equal weighting across component domains	AUC 0.89	High discrimination preserved
Threshold sensitivity 1	Severe threshold ≥ 45	|Wits|, SN-MP, asymmetry index, airway area remained significant	No directional change in main predictors
Threshold sensitivity 2	Severe threshold ≥ 55	|Wits|, SN-MP, asymmetry index, airway area remained significant	Findings robust to stricter severe cut-point
Collinearity check	Variance inflation factors	VIF range 1.34–2.41	No evidence of problematic multicollinearity
Interaction analysis	|Wits| × SN-MP; |Wits| × asymmetry; SN-MP × asymmetry	All interaction *p*-values > 0.10	No material interaction retained

## Data Availability

The de-identified data supporting the findings of this study are not publicly deposited because of ethical and privacy restrictions. They may be made available to the editorial office for confidential verification and, where ethically permissible, to qualified researchers upon reasonable request to the corresponding author.
